# Integrated 3D Hydrogel Waveguide Out-Coupler by Step-and-Repeat Thermal Nanoimprint Lithography: A Promising Sensor Device for Water and pH

**DOI:** 10.3390/s18103240

**Published:** 2018-09-26

**Authors:** Achille Francone, Timothy Kehoe, Isabel Obieta, Virginia Saez-Martinez, Leire Bilbao, Ali Z. Khokhar, Nikolaj Gadegaard, Claudia Delgado Simao, Nikolaos Kehagias, Clivia M. Sotomayor Torres

**Affiliations:** 1 Catalan Institute of Nanoscience and Nanotechnology (ICN2), CSIC and BIST, Campus UAB, Bellaterra, 08193 Barcelona, Spain; timothy.kehoe@gmail.com (T.K.); claudia.delgado@eurecat.org (C.D.S.); nikos.kehagias@icn2.cat (N.K.); clivia.sotomayor@icn2.cat (C.M.S.T.); 2 Tecnalia Research & Innovation, Mikeletegi 2, E-20009 San Sebastián, Spain; isabel.obieta@tecnalia.com (I.O.); vsaez@imasmed.com (V.S.-M.); leire.bilbao@tecnalia.com (L.B.); 3 Division of Biomedical Engineering, University Glasgow, Rankine Building, Glasgow G12 8LT, UK; a.z.khokhar@soton.ac.uk (A.Z.K.); Nikolaj.Gadegaard@glasgow.ac.uk (N.G.); 4 Institucio Catalana de Recerca i Estudis Avançats (ICREA), 08010 Barcelona, Spain

**Keywords:** hydrogel, waveguide, thermal nanoimprint lithography, water sensor, pH sensor

## Abstract

Hydrogel materials offer many advantages for chemical and biological sensoring due to their response to a small change in their environment with a related change in volume. Several designs have been outlined in the literature in the specific field of hydrogel-based optical sensors, reporting a large number of steps for their fabrication. In this work we present a three-dimensional, hydrogel-based sensor the structure of which is fabricated in a single step using thermal nanoimprint lithography. The sensor is based on a waveguide with a grating readout section. A specific hydrogel formulation, based on a combination of PEGDMA (Poly(Ethylene Glycol DiMethAcrylate)), NIPAAm (N-IsoPropylAcrylAmide), and AA (Acrylic Acid), was developed. This stimulus-responsive hydrogel is sensitive to pH and to water. Moreover, the hydrogel has been modified to be suitable for fabrication by thermal nanoimprint lithography. Once stimulated, the hydrogel-based sensor changes its topography, which is characterised physically by AFM and SEM, and optically using a specific optical set-up.

## 1. Introduction

Recent developments in optical sensors include the use of new materials which exhibit a particular behaviour in the presence of a stimulus. Hydrogels have chemical groups that make them sensitive to changes in the external environment. The stimulus-responsive or “intelligent” [1] hydrogels emerged as a promising new class of materials initially with pharmaceutical applications [2], nowadays extended to many other biomedical fields [3], especially in tissue-engineered scaffolds [4]. Small changes in the environment, such as pH, temperature [5], or ionic strength, cause a reversible phase transition in the gel structure of these polymers. This sensitivity is expressed as a volume phase transition between a swollen and a collapsed state [6]. Patterning of hydrogels for chemical sensing has been described in several reviews [7,8]. One of the most used sensing mechanisms relies on optical projectors. In fact, once patterned, hydrogels can be used as diffractive optical sensors [9,10]. A typical setup for hydrogel-based optical sensors was reported by Ye et al. [9]. It consists of a laser source, the hydrogel sensor placed near to the specimen to be studied, and a detector (photodiode) facing the latter. A similar configuration also based on a single grating structure was reported by Mao et al. [10]. In both cases, the working principle is the same: light is directed to the sensor and as it passes through it, it is diffracted into multiple diffraction orders detected by free-space optics. The expansion or change in shape of the grating lines of the hydrogel on absorption of water results in a detectable change in the intensity of the transmitted diffraction orders. By transducing the change of volume on absorption of water into an optical signal, it is possible to obtain a variation in signal of at least one order of magnitude. The disadvantage of these sensors is that the setup cannot be integrated on a chip and thus is not suitable for miniaturisation to make them compatible with other optical or microfluidic components, or for incorporation in lab-on-a-chip type devices or hand-held devices. Several realisations of hydrogel optical sensors based on three-dimensional (3D) gratings have been demonstrated, including colloidal photonic crystals [11], inverse opals [12], and holograms [13]. All of them require a complex fabrication process or design. Another type of hydrogel optical sensor is based upon a waveguide design. As presented in the patent WO2011157767A1 [14], a part of the cladding layer of an optical waveguide may be replaced by hydrogel material. The hydrogel is functionalised to immobilise analyte molecules. Their presence in the cladding layer affects the propagation of the light in the waveguide by interaction with the evanescent field, resulting in a change in speed of propagation of the light. This design requires a reference waveguide to detect the change in light speed in the sensing waveguide, as a phase shift relative to the reference beam. Other examples of waveguide sensor design are those presented in the patent EP2088423A1 [15] and by Liu et al. [16]. In the former, an optical core made of hydrogel is functionalised to immobilise analyte molecules. In the latter, a layer of hydrogel is employed to replace a portion of the cladding around a fibre Bragg grating (FBG). An advantage of the mentioned waveguide-based sensors is that they can be integrated into optical waveguiding circuits for efficient device packaging and miniaturisation. However, the optical mechanisms by which a chemical or physical change is transduced into an optical signal are relatively complex, requiring either the measurement of optical phase by recombining light with a reference beam, the use of fluorescent markers, or spectroscopic analysis of the transmitted light. Furthermore, the device structures are hybrid, composed of polymer or glass with hydrogel materials, which leads to complex fabrication processes.

The work presented in this paper solves the problems discussed above. It combines the design simplicity and a large dynamic range of a diffractive sensor with an integratable waveguide out-coupler. This 3D hydrogel-based sensor is fabricated using a thermal nanoimprint lithography (NIL) and is capable of detecting more than one environmental change, in particular, water and pH.

Previously, Ye et al. [17] used NIL to fabricate a hydrogel-based sensor, based only on a two-dimensional (2D) diffraction grating structure. Furthermore, in that case, the sensor was able to detect only variation of pH. The fabrication process employed a soft stamp with low applied pressure and it was not up-scaled in step-and-repeat mode several times on the same substrate, as we did, using a manufacturing process with the potential for high-volume and low-cost production [18]; the substrate was not silicon, and finally, the guided light was transmitted perpendicularly to the plane of the grating.

## 2. Materials and Method

### 2.1. Material Formulation

The hydrogel-based material presented in this work was based on a tert-polymer PEGDMA-NIPAAm-AA: Poly(Ethylene Glycol DiMethAcrylate-co-N-IsopropylAcrylAmide-co-Acrylic Acid) and was based on previous UV-curable formulations [19]. This stimulus-responsive hydrogel was sensitive to pH, due to the carboxylic groups of the acrylic acid, and to water, due to the 3D crosslinked structure of the hydrogel. The added thermal initiator was AIBN (Azo(bis)IsoButyroNitrile) in a concentration of 1% *w*/*w*, the cross-linker was EGDMA (Ethylene GlycolDiMethAcrylate) in 5% *w*/*w*, and the curing reaction accelerating agent was TEMED (N,N,N′,N′-TEtraMethylEthyleneDiamine) in a concentration of 0.5% *w*/*w*. The NIPAAm was dissolved in an AA-PEGDMA solution, avoiding the introduction of another solvent. To be suitable for the nanoimprint lithography process, the PEGDMA-NIPAAm-AA required the following ratio for its elements: 10:40:50 [20]. This formulation was diluted in ethanol at 30% *w*/*w*. The materials were all purchased from Sigma–Aldrich (Schnelldorf, Germany) PEGDMA (Mw 550, 98%), AIBN, EGDMA (Mw 198, 98%), and TEMED (Mw 116, 99%) were used without further purification. NIPAAm (Mw 113, 97%) was purified by recrystallization in n-hexane and AA (Mw 72, 99%) was de-inhibited using an ion-exchange resin.

### 2.2. Substrate Preparation

Silicon wafers with 400 nm thermal oxide layer and 100 mm diameter (supplied by Silicon Materials Inc., Glenshaw, PA, USA) were used as substrate, because the thermal oxide is a dielectric layer with a lower refractive index *n* (*n* = 1.456 [21]) compared to the hydrogel (*n* = 1.498 [22]). This is an important requirement to ensure that the light is guided through the hydrogel. To achieve a good adhesion and wettability of the hydrogel to the selected substrate, the SiO_2_/Si substrates were coated with a TPM (3-(Trichlorosilyl)Propyl Methacrylate) layer [23]. TPM (Sigma-Aldrich, Schnelldorf, Germany) was first diluted in a solution of heptane:carbon tetrachloride (4:1) and then the substrates were submerged in the final solution at room temperature for 5 min in an N_2_ atmosphere. Finally, the substrates were washed with hexane and water.

### 2.3. Stamp Manufacturing

**Figure 1:**
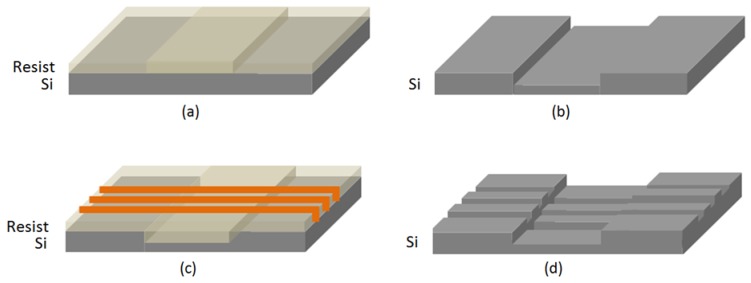
Schematic of the process flow for the 3D silicon master stamp fabrication. (**a**) UV photolithography exposure into a photoresist to define the waveguide area; (**b**) RIE to transfer the pattern into silicon (Si); (**c**) electron beam lithography to define the out-coupling grating-structures; (**d**) etching of Si waveguides. PMMA (positive tone) is used as a mask during the e-beam exposure process.

A two-step process was necessary for the manufacturing of the 3D silicon master stamp, illustrated in Figure 1. The first step consisted of UV photolithography exposure into a photoresist to define the waveguide area (Figure 1a) followed by reactive ion etching (RIE) (Figure 1b) to transfer the pattern into silicon. The second step consisted of electron beam lithography exposure to define the out-coupling grating-structures (Figure 1c), followed by silicon etching (Figure 1d). PMMA (positive tone) was used as a mask during the e-beam exposure process. Dry etching was performed using a mixture of SF6/C4F8 gases (25/45 sccm).

### 2.4. Patterning Method

**Figure 2:**
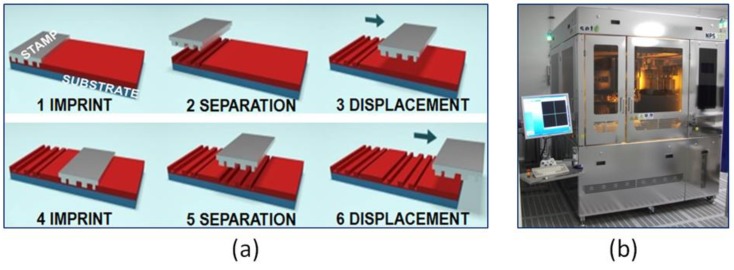
(**a**) Step-and-repeat NIL process. (**b**) NPS 300 stepper.

The sensor was fabricated by means of the thermal NIL process [24] as a high-resolution and high-throughput lithography technique. NIL is based on the mechanical deformation of a resist layer with a stamp presenting a surface topography to be replicated. It is a relatively simple process, and in this work, we demonstrated that it is suitable to fabricate a 3D hydrogel-based sensor. The sensor was fabricated using the thermal NIL process in a step-and-repeat mode using the NPS 300 stepper (SET) as imprinting tool, as shown in Figure 2b. After imprinting a first die, the stamp was released from the substrate, displaced to the next die, put again into contact with the substrate, and thermally imprinted (Figure 2a).

### 2.5. Sensor Evaluation Setup

The optical response of the imprinted sensor was measured in the unswollen and swollen state in the presence of water using a purpose-designed optical setup. In this setup, the light came from a diode-pumped solid-state laser at 488 nm (Spectra-Physics Low Power CW 488 nm Lasers-Cyan™ Scientific (100 mW output power). It was first coupled to a single-mode optical 3 μm core tapered fibre through a microscope objective lens and then coupled from the tapered end of the fibre to the waveguide. Micrometer-driven positioning X Y Z stages were used for accurate alignment of the coupled light. The out-coupled light was detected using a miniature digital microscope with a CCD sensor.

## 3. Results and Discussion

### 3.1. Device Concept

**Figure 3:**
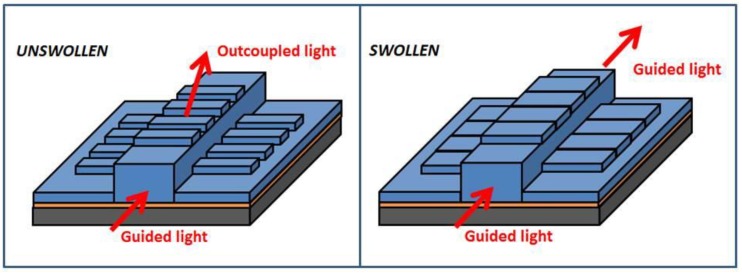
Schematics of the device based on a grating on waveguide. A relative change in dimensions occurs in the grating in the presence of an external environmental change. Two states are identified (unswollen and swollen) related to the behaviour of the selected hydrogel material. In the presence of an environmental change (water or pH), the hydrogel reaction leads to a morphological change of the grating structure.

The device was based on a grating structure formed on the upper part of a waveguide; the grating structure was extending in the perpendicular direction to the light propagation direction, which will be referred to as the direction of the waveguide. The grating and the waveguide were made of the same material, which was based on a hydrogel formulation. The prototype device had two well-defined states: the unswollen and the swollen states. In the unswollen state, the guided light was scattered from the coupled grating structures, making it optically visible when viewed from the top. In the swollen state, the perturbation effect was eliminated as the elements of the grating structures were brought in intimate contact, allowing the light to be guided through the waveguide, becoming invisible when viewed from the top. Figure 3 illustrates the relative change in dimensions occurring in the grating in the presence of an external environmental change. The two states are related to the behaviour of the selected hydrogel material. In the presence of an environmental change (water or pH), the hydrogel reaction leads to a morphological change of the grating structure.

The extension of the grating into the planar area beside the waveguide is a feature of the fabrication process. The devices are intended to be cost-effective, and so the fabrication process is designed to be efficient, so that an effective device may be fabricated with the least amount of process steps. It would be possible to apply the grating nanoscale patterning only to the waveguide structure by using more sophisticated overlay alignment of the optical lithography and electron-beam lithography processes, but this would require a greater investment of time and resources in the fabrication process. The most cost-effective process is to cross an array of microscale waveguides with extended nanoscale gratings. Importantly, this does not affect the designed optical behaviour of the device. If light is efficiently in-coupled to the waveguide, then out-coupling occurs only along the waveguide ridge. However, if there is poor in-coupling, leading to waveguiding through the plane of the hydrogel, then out-coupling can also occur in the planar region, which would have an impact on the detected signal level, even though it follows the response behaviour of the device to external stimuli. The solution is to ensure efficient coupling of light from the source into the waveguide. It would be necessary at a later device prototyping stage to assess the cost of achieving precision for in-coupling from an integrated light source, and compare to the cost of greater precision in overlay of the patterning steps (to limit the grating to the top surface of the waveguide), as part of a complete production process cost analysis. 

### 3.2. Device Fabrication

**Figure 4:**
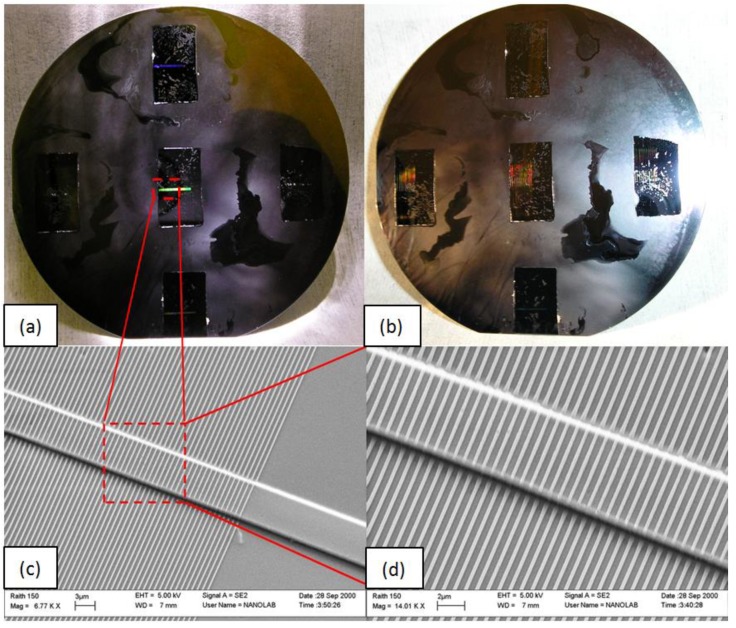
Multiple imprinted dies containing the sensor devices over 4 inch silicon wafer. (**a**,**b**) refer to the same wafer: in (**a**), the light applied through a camera is diffracted in the grating area of each die. The waveguide lines can be observed diffracting in (**b**). (**c**,**d**) Top view scanning electron microscopy images of the structures imprinted in the thermally curable hydrogel (**a**,**b**), where (**d**) gives a magnified view of (**c**).

Although NIL has demonstrated its flexibility and low cost of ownership, the high cost for the master stamp generation remains a barrier. To increase the lifetime of the master stamp, it is necessary to enhance its anti-adhesion properties. Several different anti-adhesion layers were deposited on the silicon master stamp surface (containing the negative topography of the sensor to be imprinted) and the relative lifetime was studied in terms of number of imprints. Each imprint was performed by step-and-repeat thermal NIL and the followed process parameters are detailed later in this paragraph. Four different commercial anti-adhesion materials were tested: OctadecylTrichloroSilane (OTS) [25] from Sigma-Aldrich (Schnelldorf, Germany), 1,1,2,2-tetrahydrooctylTriChloroSilane (F13-TCS) [26] from ABCR (Karlsruhe, Germany), Optool™ DSX from Daikin Chemical Europe (Düsseldorf, Germany) and Fluorolink^®^ S10 [27] from Solvay Specialty Polymers (Bollate, Italy). The process deposition of each material can be found in the respective reference. OTS was found to be the anti-adhesion material with the longest lifetime, allowing up to ten imprints, in contrast to the three imprints possible with the other ones. The maximum number of imprints was not limited by the OTS. In fact, the limit was not explored. Before each imprint, the hydrogel formulation was manually drop-dispensed on the substrate using a 3 μL syringe. Upon touching the substrate, the resist immediately spread, showing good wettability. However, the resist underwent a colour change when left on the substrate for more than 4 min prior to imprinting due to the fast evaporation of ethanol. Thus the time elapsed between drop dispensing and imprinting needed to be within seconds. The imprinting parameters were optimized to achieve a short cycle time and good reproducibility. The best imprinting conditions found to perform the step-and-repeat thermal NIL process were: 200 °C as imprinting temperature, 1 min as imprinting time, 100 N as imprinting force, 40 °C as separation temperature. Working within this time scale, we successfully demonstrated multiple imprinted dies containing the sensor device over a 4 inch silicon wafer, as illustrated in Figure 4a,b. The imprinted structures were then characterised by scanning electron microscope (Figure 4c,d). Good replication fidelity was observed between the silicon master stamp structures and those imprinted into the resist, confirmed by a variation of less than 2% in the critical dimensions of the waveguide geometries.

A possible improvement to the device fabrication process could come through the development of a hydrogel formulation that could be deposited by spin-coating on the whole wafer. Furthermore, it could be possible to reduce the curing time (below 1 min), applying a higher temperature, or exploring the addition of a different thermal initiator. 

The critical dimensions of the waveguide and the grating in the silicon master stamp are summarized in Table 1. These dimensions were optimized using Finite Difference Time Domain numerical simulations (RSoft Design Group Inc., Ossining, NY, USA), so that the maximum intensity of light was coupled out of the waveguide, at an angle of approximately 65° to the plane of the waveguide.

### 3.3. How Water Affects the Sensor

**Figure 5:**
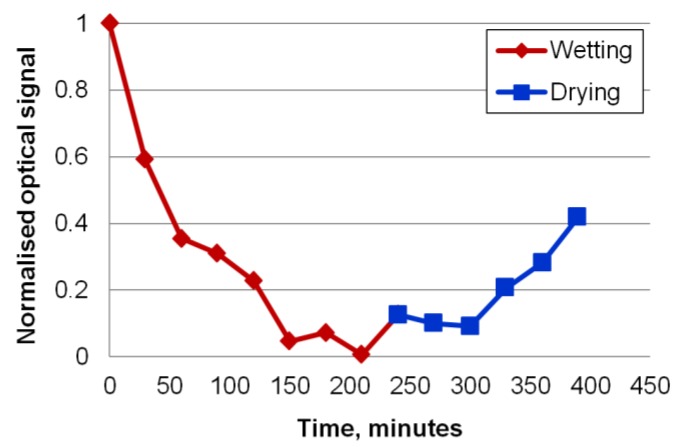
Normalised optical response of the sensor to water.

The optical response of the imprinted sensor was observed in the unswollen and swollen state. Figure 5 shows the variation of the normalised intensity of the measured light directed out of the hydrogel layer when the hydrogel absorbs water and expands. Such variation in signal intensity improves sensing resolution, and enables two distinguishable states to be defined, indicating the absence or presence of the chemical or physical state being detected. The intensity of the light emitted perpendicular to the surface at room temperature (20 °C) and ambient humidity (approximately 40%) is normalised to the value of 1.

To generate the graph, a drop of distilled water was deposited on the sample to induce water absorption in the hydrogel as a test of the dynamic range of the material and optical design. Excess moisture was removed from the sample surface at intervals of 30 min by blowing with air and the optical signal measured; about 1 min was occurring between the removal of excess moisture and the optical signal measurement. A drop of water was then replaced on the sample to continue the process of absorption. After 210 min, the optical signal was reduced to almost zero, which represented the full expansion of the hydrogel so that the out-coupler structure was flattened. The sample was then allowed to dry in ambient air, and the optical signal returned to 0.4 of its previous dry level, showing a partial but measurable recovery. Such a response may find application as a long-term, water-detection sensor in buildings or storage facilities. For such types of application, it would be necessary to evaluate the long-term stability of the hydrogel polymer inside the 3D network that it creates after curing. Furthermore, the other optical components and their mounting and alignment should be stable over a number of years, including a diode laser light source, photodiode detector, small lens to couple light into the waveguide, and lens to collect out-coupled light for detection.

The out-coupled optical signal becomes negligible when the waveguide is fully wet and swollen. To avoid false negative responses due to some other system defect, such as misalignment of coupling from the source, for example, it should be possible to add a reference waveguide, without a grating. This would be a branch from the grating waveguide, with detection of the light transmitted through this acting as an internal system calibration. In principle, optical readout is also possible by transmission through the grating waveguide, which is increased when the device is wet. Transmission testing was not performed with the current devices due to the presence of direct illumination from the source in the detection field of view. Since the design used in the current experiments was a short linear waveguide, it was difficult to isolate guided light from direct illumination emanating from the end of the source fibre optic and passing over the surface of the waveguide. An improved design to avoid direct illumination of the sensor, for guided transmission detection, would include a bend in the waveguide through 90° in the plane, using a curved section.

**Figure 6:**
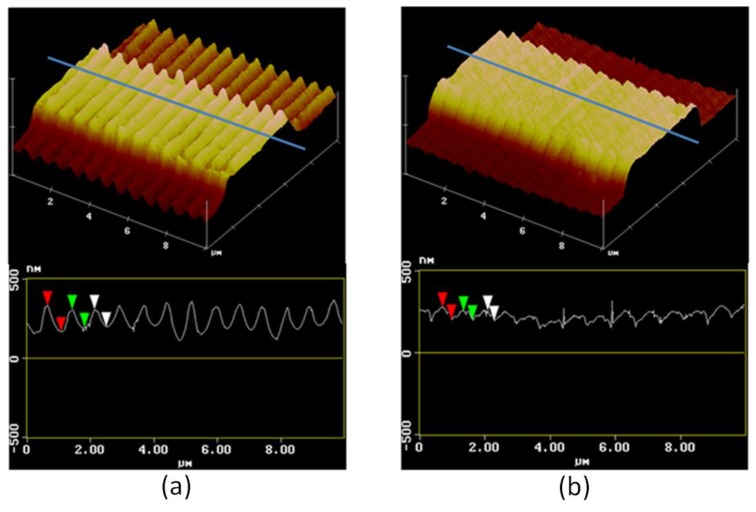
Atomic force microscope images showing the patterned device in the dry (**a**) and the wet (**b**) state. The structures in the dry state are illustrated in (**a**), their expansion with water absorption and the partial closure of the spaces between the out-coupler grating lines are illustrated in (**b**).

The topographical change between dry and wet state was characterised by atomic force microscope (AFM) measurements, as shown in Figure 6. The structures in the dry state are illustrated in Figure 6a, while their expansion with water absorption and the partial closure of the spaces between the out-coupler grating lines is illustrated in Figure 6b. The wet state topography was measured placing a droplet of water on the upper surface of the sample for two hours and then removing it by blowing air immediately before performing the AFM measurement.

### 3.4. How pH Affects the Sensor

**Figure 7:**
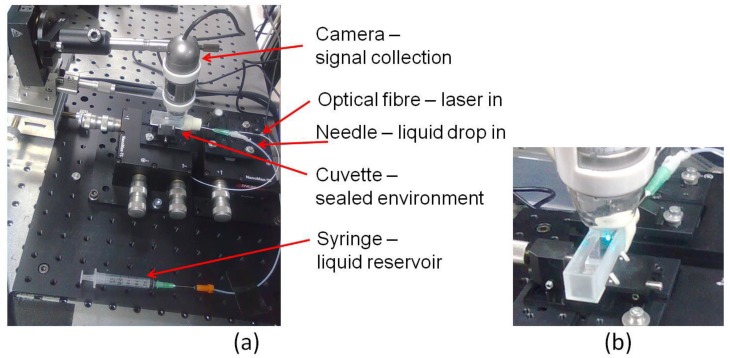
(**a**) Optical setup to test the sensor response to pH. (**b**) A magnified view of the cuvette environment.

For these measurements, the sensor device was placed in a quartz cuvette, as shown in Figure 7.

A droplet of acid solution was added to the atmosphere inside the cuvette to saturate it with acidic vapour, as shown in Figure 7a. A syringe was used as liquid reservoir for the acid solution, which was drop-dispensed inside the cuvette using a needle. The optical fibre and the inlets to provide the acid solution were connected to the inside of the cuvette via a rubber septum. A magnified view of the cuvette environment is given in Figure 7b.

**Figure 8:**
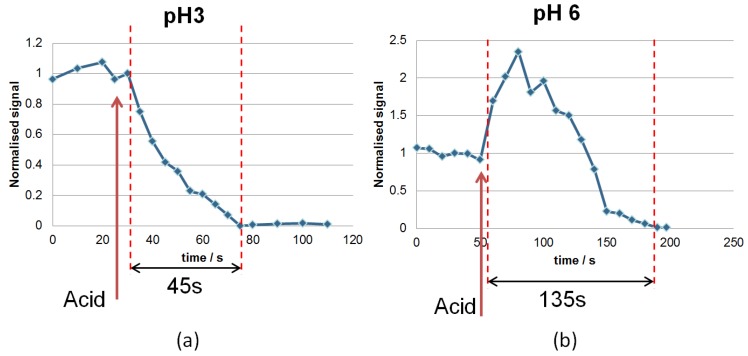
Optical signal response to the introduction of acid of pH 3 (**a**) and pH 6 (**b**). Initially, the sample was at neutral pH and dry, in equilibrium with the humidity in the room.

Two different pH solutions (pH 3 and pH 6) were tested to evaluate the device reaction to this change in external environment, as shown in Figure 8. The expected swelling from Acrylic Acid (AA) with pH between 4 and 5 was tested in bulk and in correlation with a pH of 4.7. Both pH tests resulted in a notorious swelling. Initially, the sample was at neutral pH and dry, in equilibrium with the humidity in the room. Drops of acid were added successively in two steps from pH 6 to pH 3. In between measurements at one pH level, the waveguide sample was removed from the cuvette for one hour and dried by blowing nitrogen over it to return it to a nonswollen state. An increase in signal was observed during drying, as soon as drying began, and at the same rate as the signal reduced after the addition of a droplet of acid solution. However, the measurement was not continued until the signal was fully restored. The cuvette and tubing for the acid were rinsed with DI water. Acid was added at time t = 50 s for pH 6 and t = 25 s for pH 3. Immediately after the addition of a drop of acid, the optical signal changed due to swelling of the waveguide and out-coupler with absorption of moisture. The swelling behaviour was greatly accelerated by the presence of acid compared to the response in the presence of water alone (shown in Figure 5). The reason for this behaviour is that changes in pH provoke a movement of ions, which have to get into the nanoporous structure of the polymer chains; in presence of water, the water molecules have to get into the nanoporous structure of the polymer chains. Ions are smaller and move faster into the nanostructure than the water molecules, which are much bigger and move more slowly. At pH 3, there was gradual swelling, probably due to the humidity supplied to the system by the vapour, whereas at pH 6 an increase in the normalized light measured could be observed with a peak doubling in intensity. This behaviour was detected a few seconds after adding the drop of solution; afterwards, the signal decreased and became undetectable. The transient region increasing before reducing may be due to a temporary increase in grating line width as the smaller structures absorb water first. After absorption by the larger waveguides, the whole structure became smooth and the out-coupling reduced to zero. Another contribution to the increase in the normalized light measured in the case of pH 6 may be related to the hydrogel material configuration in the 3D network. In particular, the long polymer chains that form the gel take some time to rearrange themselves in response to change in pH. As a consequence, a few seconds later, still more output was observed.

The sensor was able to detect the change in gel response to change of pH, near the pKa of 4.7 of the acrylic acid groups. There is an equilibrium state due to the presence of water (humidity sensitivity, neutral pH of water) and a second equilibrium state can be reached when the pH of the aqueous solution is changed externally (from neutral or alkali to acid). The only way of discriminating between both is with an additional capability of the sensor to measure the ionic strength of the media. A hydrogel is responsive to pH due to sidegroups that have a charge that will depend on their pH. As these sidegroups become charged, there must be additional counterions present to make the gel electrically neutral. These counterions, however, cause an osmotic pressure difference between the gel and the solution. As a result of this osmotic pressure, the gel will take up solvent and swell until the elastic forces in the hydrogel are in equilibrium with the osmotic force [28]. This pH-dependent swelling behaviour is also dependent on one other very important parameter: the ionic strength of the solution. In the most extreme case where there is just water, only OH^−^ and H^+^ are present in the solution. When ions are present in the solution, the pH changes and it is detected, but only because the ionic strength is low enough. Finally, when a lot of ions are present in the solution (i.e., the solution has a high ionic strength), swelling is also problematic as the solution outside of the gel already has a very high osmotic pressure and the osmotic pressure inside the gel does not differ significantly from that outside the gel (explained by the Donna Theory: the size of the gel is dependent only on the osmotic pressure difference between the outside of the gel and the gel’s interior). Therefore, the pH-sensitive sensor is also sensitive to ionic strength and the ionic strength should be controlled during experiments.

**Figure 9:**
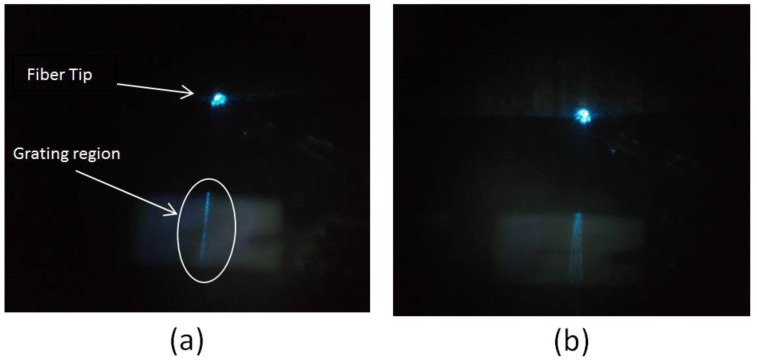
Optical microscope images of the out-coupled light from the imprinted sensor in the dry state. In (**a**), the out-coupling in the grating region is well defined, while in (**b**), a divergence of the guided wave can be observed, indicating a poor-performance sensor.

Several chips containing the imprinted structures were observed with an optical microscope, and in some of them it was possible to observe some partial waveguiding in the plane adjacent to the line of the waveguide. In Figure 9a, an example of good-quality light out-coupling from the grating region is shown, in contrast with the image shown in Figure 9b, where it is possible to note an undesirable divergence of guided wave in the plane adjacent to the grating, which is related to the presence of defects after the patterning step (such as sidewalls with rounded profile, or incomplete filling). In this case, blue spots of lights appear as a consequence of partial scattering along waveguide lines.

## 4. Conclusions

A thermally curable hydrogel based on the combination of PEGDMA, NIPAAm, and AA has been formulated and optimized for processing by thermal nanoimprint lithography (NIL). This stimulus-responsive formulation is demonstrated to be sensitive to water and to pH. It was nanostructured using thermal NIL in step-and-repeat mode, thus showing the potential for high-volume and low-cost production. The imprinted samples are 3D sensor devices based on a grating on waveguide. The sensor presented here solves the problems of hydrogel-based sensors found in the literature (complexity in the fabrication process, in the design, in the miniaturisation, in the signal detection) by combining the simplicity of design, a large dynamic range of diffractive sensing, and the suitability for integration of a waveguide out-coupler. The device fabricated by the NIL process can be integrated in a chip to form a sensor in a purpose-designed circuit.

There are a range of possible integrated readout configurations that would be practical for a finished device. For example, mounting of a photodiode with a small collection lens above the grating could be achieved with some polymer spacer elements to position the detector at the appropriate height above the hydrogel chip. Transmission readout, with the possible inclusion of a reference waveguide channel without a grating section, would make the overall signal analysis more robust and decouple the measured sensor signal from possible system variations, such as changes in coupling efficiency or light source output intensity. As discussed above, this should ideally also include a 90-degree turn of the waveguide in the plane. Since the dimensional changes in the imprinted hydrogel structures have been successfully demonstrated, it should be possible also to reverse the direction of the optics, and to design a grating for coupling of light into the waveguide from above the plane, with detection of the transmitted light at the output end of the waveguide. Such a design might have advantages in reducing the design tolerances for alignment of the source to the waveguide.

## Figures and Tables

**Table 1:** Dimensions of the waveguide and grating structures in the silicon master stamp.

Parameter	Waveguide Width	Waveguide Height	Grating Height	Grating Line Width	Grating Period
Value	1–5 μm	0.5–1.0 μm	100–150 nm	400–500 nm	700–800 nm
